# Molecular characterisation of virus in the brains of patients with measles inclusion body encephalitis (MIBE)

**DOI:** 10.1186/1743-422X-10-283

**Published:** 2013-09-12

**Authors:** Diana R Hardie, Christine Albertyn, Jeannine M Heckmann, Heidi EM Smuts

**Affiliations:** 1 Division of Medical Virology, Department of Clinical Laboratory Sciences, University of Cape Town and National Health Laboratory Service, Cape Town, South Africa; 2 Division of Neurology, Department of Medicine, University of Cape Town, Cape Town, South Africa

**Keywords:** Measles inclusion body encephalitis, MIBE, Subacute measles encephalitis, Neuro-virulence, Mutation, Immuno-compromised, Human immunodeficiency virus

## Abstract

**Background:**

During 2009/10 a major measles epidemic caused by genotype B3 occurred in South Africa. Measles inclusion body encephalitis (MIBE) was diagnosed in a number of highly immuno-compromised HIV patients. The diagnosis was based on typical clinical and MRI findings and positive measles virus PCR in brain or CSF.

To characterize the brain virus, nucleoprotein, matrix, fusion and haemagglutinin genes from 4 cases was compared with virus from acutely infected patients.

**Methods:**

cDNA was synthesized using random primers and viral genes were amplified by nested RT-PCR. PCR products were sequenced in the forward and reverse direction and a contig of each gene was created. Sequences were aligned with reference sequences from GenBank and other local sequences.

**Results:**

Brain virus was very similar to the South African epidemic virus. Features characteristic of persistent measles virus in the brain were absent. Mutation frequency in brain virus was similar to epidemic virus and had the same substitution preference (U to C and C to U). The virus of 2 patients had the same L454W mutation in the fusion protein.

**Conclusion:**

The brain virus was very similar to the epidemic strain. The relatively few mutations probably reflect the short time from infection to brain disease in these highly immuno-compromised patients.

## Background

During 2009 and 2010 a widespread measles virus epidemic occurred in South Africa. More than 18 000 cases were laboratory confirmed. The majority of infections were in young people. One third were infants less than one year of age and the rest were between one and 40 years [1]. This outbreak occurred in a population with a very high HIV prevalence. South Africa has an average HIV prevalence of 30.2% in women attending antenatal clinics and an estimated prevalence in all adult South Africans of 17.9% (15–49 years) [2]. During the course of this epidemic, a high rate of complications was seen in HIV-infected subjects and a number of patients developed a distinct neurological syndrome, confirmed to be measles inclusion body encephalitis (MIBE). The first 8 confirmed cases [3] were all young (under 40 years), HIV-infected and had low CD4 counts. Most gave a history of measles in the preceding weeks. MIBE was not identified in any HIV- negative individuals during this epidemic.

There are three neurological complications following acute measles infection. Within 2 weeks of the onset of the rash, an acute demyelinating encephalomyelitis (ADEM) may develop. This is an auto-immune phenomenon as measles virus is not present in the brain [4]. Measles inclusion body encephalitis, also termed subacute measles encephalitis, typically occurs one to nine months after acute measles infection in highly immuno-compromised individuals, either as a result of HIV infection or haematological malignancies [5,6]. Sub-acute sclerosing pan encephalitis (SSPE) typically occurs in apparently immuno-competent persons, but symptoms develop only after a prolonged latent period and the virus from brain tissue is highly defective. In both MIBE and SSPE the brain is the site of on-going measles pathology. The virus evades immune defenses by spreading from cell to cell within the brain [7]. Key viral structural genes (matrix, fusion and haemagglutinin) are highly mutated, rendering the proteins nonfunctional [8]. We were interested to investigate the virus from brains of local MIBE cases to determine whether the virus was similarly defective and whether there were any common features which could explain the pathogenesis of the condition. Accordingly, the nucleoprotein (N), matrix (M), fusion (F) and haemagglutinin (H) genes from 4 MIBE cases (see Table 1 for clinical details) were compared with virus from patients acutely infected during this epidemic.

**Table 1:** Details of study patients

	**Patient 1**	**Patient 2**	**Patient 3**	**Patient 4**
Age	26	14	34	24
Sex	F	M	F	F
CD4 count	65	1	26	11
Acute measles prior to disease onset	3 months	Unknown (no rash)	3 weeks	4 months
Sample type analysed	Brain	CSF	Brain	Brain
Vaccine history	Unknown	Received measles vaccine 4 months previously*	Unknown	Unknown

*Schwartz strain.

## Results

Complete sequence data for N, M, F and H genes was obtained for brain virus of 2 patients (1 and 3) and epidemic virus from 6 patients (GenBank accession numbers KC305651-KC305689]. Incomplete sequence data was obtained from brain virus of a further 2 patients (2 and 4). Partial sequence was due to limited clinical material available for PCR amplification. For patient 4, most of the H gene sequence was available (nucleotides 246–1874) and partial sequence for the F gene (nucleotide 1–1690) and for patient 2, the complete N, M and F genes were sequenced.

### Phylogenetic analysis of epidemic virus

**Figure 1:**
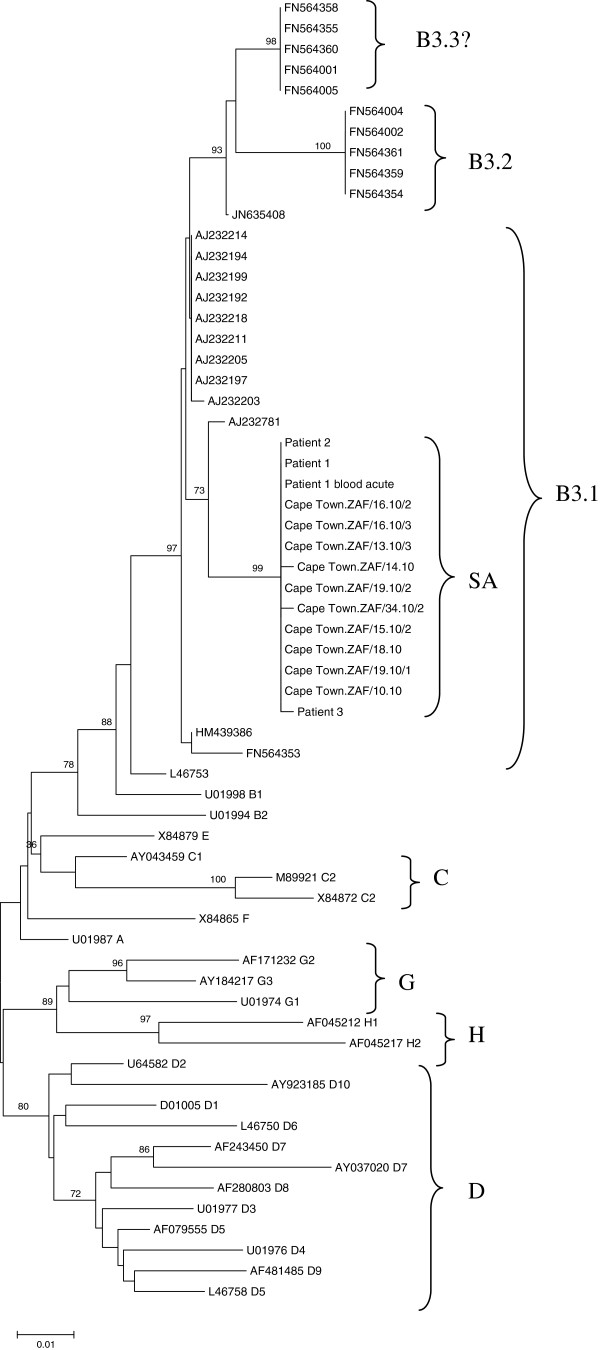
**Phylogenetic tree generated by neighbour-joining analysis of the 3′ hypervariable region of the nucleoprotein gene of measles virus from patients with acute measles infection and MIBE during the measles epidemic of 2009–2010 in South Africa.** Reference sequences of other measles virus genotypes were retrieved from the NCBI GenBank database and are indicated by accession numbers. Bootstrap values greater than 75% are indicated at the nodes of the tree. The branch lengths are proportional to the evolutionary distances as shown on the scale.

**Figure 2:**
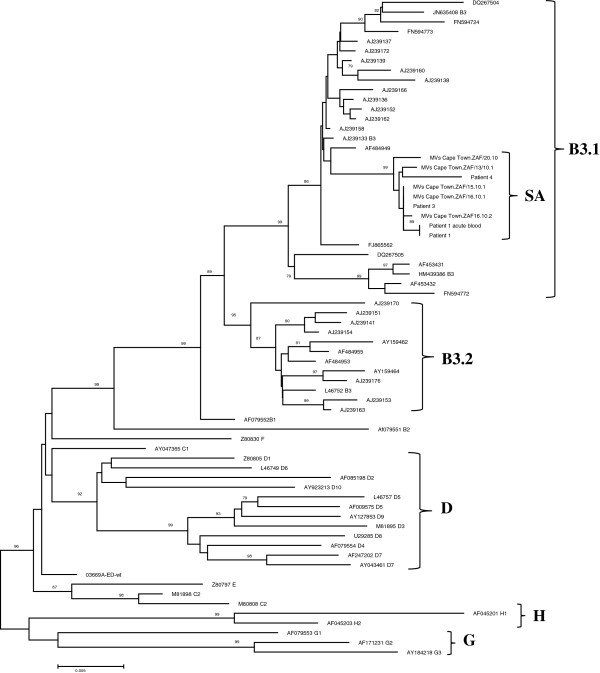
**Phylogenetic tree generated by neighbour-joining analysis of the haemagglutinin gene of measles virus from patients with acute measles infection and MIBE during the measles epidemic of 2009–2010 in South Africa.** Reference sequences of other measles virus genotypes were retrieved from the NCBI GenBank database and are indicated by accession numbers. Bootstrap values greater than 75% are indicated at the nodes of the tree. The branch lengths are proportional to the evolutionary distances as shown on the scale.

**Figure 3:**
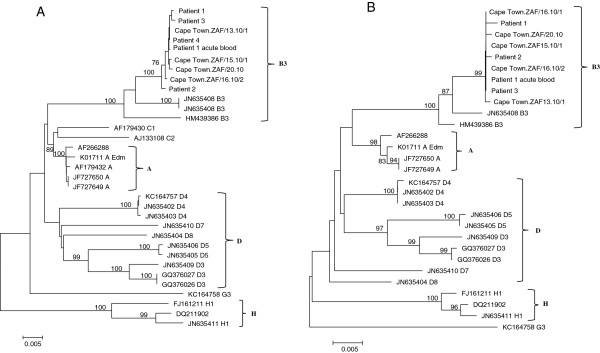
**Phylogenetic trees generated by neighbour-joining analysis of the fusion (A) and matrix (B) genes of measles virus from patients with acute measles infection and MIBE during the measles epidemic of 2009–2010 in South Africa.** Reference sequences of other measles virus genotypes were retrieved from the NCBI GenBank database and are indicated by accession numbers. Bootstrap values greater than 75% are indicated at the nodes of the tree. The branch lengths are proportional to the evolutionary distances as shown on the scale.

Phylogenetic analysis of the nucleoprotein and haemagglutinin genes confirmed that the South African epidemic in 2009/10 was caused by genotype B3. (Figures 1, 2) The South African sequences formed a distinct cluster within genotype B3.1 and this held true in phylogenetic trees of the F and M genes as well (Figure 3A and B). Brain and acute virus sequences clustered closely together on all phylogenetic trees (Figures 1, 2 and 3).

**Figure 4:**
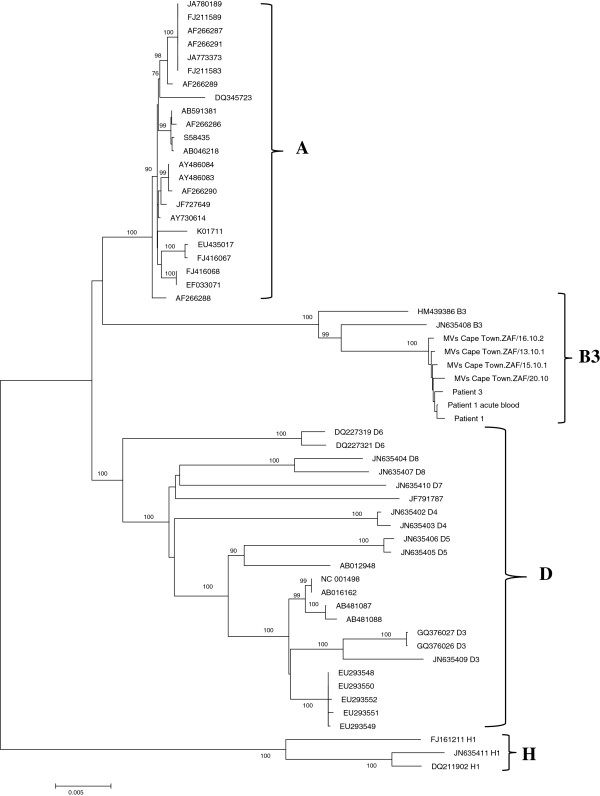
**Phylogenetic tree generated by neighbour-joining analysis of concatentated nucleoprotein, matrix, fusion and haemagglutitin genes of measles virus from patients with acute measles infection and MIBE during the measles epidemic of 2009–2010 in South Africa.** Reference sequences of other measles virus genotypes were retrieved from the NCBI GenBank database and are indicated by accession numbers. Bootstrap values greater than 75% are indicated at the nodes of the tree. The branch lengths are proportional to the evolutionary distances as shown on the scale.

BLAST analysis of the South African consensus M and F genes showed that the epidemic virus was most closely related to JN635408, a 2005 isolate from New Jersey, USA. The consensus N and H genes were more closely related to 1998 measles virus isolates from Nigeria (AJ232781 and AJ 239171). A concatenated phylogenetic tree of the full N, M, F and H genes confirmed a close relationship of the South African measles viruses to B3 viruses from North Africa (Figure 4).

There was very little sequence variation in the epidemic virus. A total of 24 polymorphisms were present in the six acutely infected patients over the 6.728 Kb region sequenced (giving a mutation frequency of 0.56 per 1000 nucleotides).

### Sequence analysis of brain virus

Sequences from the acute epidemic virus were aligned and used to create a South African consensus sequence to which the brain virus of patients 2, 3 and 4 were compared. The brain virus from patient 1 was compared with her own acute blood virus from 3 months earlier. In all patients the brain virus was very similar to the epidemic virus. Typical features of measles virus of SSPE/MIBE cases were not present, namely hyper mutation of the matrix and fusion proteins or truncation of C terminal end of fusion protein gene [9]. Each MIBE patient had a unique pattern of mutations in one or more of the N, M, F and H genes (Table 2). In total (for all genes sequenced) there were 18 polymorphisms present in brain virus relative to the South African consensus sequence, (Table 2).

**Table 2:** Nucleotide and amino acid differences in the nucleoprotein (N), matrix (M), fusion (F) and haemaglutinin (H) genes between the South African measles consensus B3.1 sequence and brain virus from 4 patients with MIBE

**Gene**	**Nucleotide position**	**Nucleotide**					**Amino acid position**	**Amino acid**				
		**SACon**	**Pat1**	**Pat2**	**Pat3**	**Pat4**		**SACon**	**Pat1**	**Pat2**	**Pat3**	**Pat4**
N	572	A	A	A	**G**	n/d	174	I	I	I	V	n/d
	1623	U	U	U	**C**	n/d	524	L	L	L	P	n/d
												
M	328	U	**C**	U	U	n/d	99	I	T	I	I	n/d
	388	U	**C**	U	U	n/d	119	L	P	L	L	n/d
	399	A	A	**U**	A	n/d	123	T	T	S	T	n/d
F	105	C	C	**U**	C	C						
	127	C	C	**G**	C	C						
	162	C	C	**U**	C	C						
	203	C	C	C	C	**U**						
	323	G	G	G	**C**	G						
	331	C	C	**A**	C	C						
	461	G	G	**C**	G	G						
	766	U	U	U	**C**	U						
	1483	G	G	G	G	**A**						
	1592	A	**C**	A	A	A	337	M	L	M	M	M
	1944	U	**G**	U	**G**	n/d	454	L	W	W	W	n/d
H	1362	C	**U**	n/d	C		448	R	C	R	R	R
	1742	C	**U**	n/d	C	C						

*SACon* South African consensus sequence, *Pat1* patient1, *Pat2* patient2, *Pat3* patient3, *Pat4* patient4, *n/d* not done. Bold font indicates nucleotide that varies from consensus. Nucleotides 105 to 461 in the fusion gene are non coding.

U to C and C to U mutations were the most common mutations present in the epidemic virus and were also the most frequent in the brain virus. The calculated mutation frequency in brain virus was 0.87 per 1000 nucleotides. This was not significantly different from the mutation frequency of the epidemic virus which was 0.56 per 1000 nucleotides (p=0.19). Interestingly, of the 10 substitutions that occurred in coding regions in the brain virus, 8 were non-synonymous. The most variable region was the non coding region of the fusion gene.

Patients 1 and 3 had an identical U to G mutation at position 1944 in the F gene which gave rise to a L454W substitution in the fusion protein. Of note, the L454W substitution was not present in the blood virus of patient 1 collected during acute measles infection 3 months earlier.

## Discussion

The measles virus outbreak in South Africa was due to genotype B3 a known African genotype. It was probably introduced into South Africa from the north where ongoing circulation of measles virus of this genotype has been documented in various African countries for many years [10,11]. Infection spread widely in the South African population due to poor herd immunity. Analysis of measles virus sequences from acutely infected individuals showed that there was a low level of genetic variability in the epidemic virus. This is typical of a single source introduction which is followed by dissemination in a non immune population [12,13].

While invasion of the brain during acute measles may occur [14], MIBE is normally very rare. Most highly immuno-compromised patients who had acute measles in this epidemic did not develop MIBE. Clearly both viral and host factors play a role in the disease process. While much is known about the genetic characteristics of SSPE virus, this is not the case for MIBE virus. We were interested to sequence the brain virus in some of these cases to determine its similarity to the epidemic virus in order to try to gain insight into the pathogenesis of this normally very rare condition.

On the whole, the brain virus was very similar to the acute epidemic virus. This is in contrast to what is found with SSPE [9,15] and also what has previously been reported for MIBE [15]. Mutation rates were similar in brain (0.87 per 1000 bases) to the epidemic virus (0.56 per 1000 bases). However, of the 10 substitutions that occurred in the coding regions of genes from brain virus, 8 were non-synonymous (in comparison to only 4 of 22 from the epidemic virus). This could imply either that some selection pressure was acting on the virus in the brain or that a mutation generating process was operating.

Measles virus does not usually replicate extensively in brain tissue and it is thought that key mutations may be needed to confer a neuro-virulent phenotype [16,17]. Because of the many mutations found in SSPE virus, it is difficult to determine which are responsible for neuro-tropism and which are merely the consequence of mutations accumulating in genes which are no longer essential for virus replication in the brain. By studying the fewer mutations in virus from MIBE brains it may be possible to determine which were responsible for the gain in neuro-tropism. In this study, most of the mutations in the brain virus were different for each patient and targeted different genes. However, one point mutation in the fusion protein, namely L454W was present in 2 patients (1 and 3). It is unlikely to have been a chance polymorphism in some circulating virus because it was not present in the acute measles sequence of patient 1. Both leucine and tryptophan are neutral, non-polar amino acids. However, this substitution is not favoured, especially in a membrane protein, and the substitution would be likely to change the properties of the protein [18]. This substitution falls in the extra-cellular domain of the fusion protein, adjacent to the heptad repeat B domain (HRB). Ayata *et al*. [17] showed that a single substitution (T461I) was responsible for neuro-virulence of an SSPE strain in a hamster model. This region is thought to interact with the as yet unknown measles virus receptor in the brain and plays a role in the fusion process. The independent presence of this mutation in 2 patients is interesting.

There were remarkably few mutations in all four of the genes sequenced from the brain virus. In measles virus from persistently infected brain, both in humans as well as in animal models, the nucleoprotein gene typically retains its function as this protein is required to form intact ribonuclear protein complexes to enable the virus to move from cell to cell in the brain [7]. The matrix protein, on the other hand, is usually highly mutated as this protein is not needed for replication in the brain [19,20]. Perhaps sufficient time had not elapsed for mutations to accumulate in this gene before the clinical presentation of MIBE in our highly immuno-compromised patients. In 3 MIBE patients who gave a history of acute measles, the median time from acute infection to onset of neurological disease was about 10 weeks. In all patients there was a rapid neurological deterioration after presentation, which probably reflects the poor immune control of measles virus in the brain.

In conclusion, it is probable that host factors were largely responsible for driving the disease process. However, not all severely immuno-compromised HIV patients infected with measles developed MIBE. Viral factors also must have played a role. Intriguingly the brain virus was very similar to the epidemic virus and did not show features previously reported to be characteristic of MIBE. The mutation frequency was similar to epidemic virus, but significantly these mutations were more likely to be non-synonymous. A key finding was that 2 patients had the same L454W mutation in the fusion protein.

## Materials and methods

### MIBE patients

Patients were identified as having MIBE based on typical clinical and magnetic resonance (MRI) findings as previously described [3] and if measles virus was detected by PCR in brain tissue or cerebrospinal fluid (CSF). An in-house diagnostic nested RT-PCR assay was used to amplify a 500 nucleotide fragment of the nucleoprotein gene [13]. Measles virus positive brain or CSF was available for study on 4 patients.

Patient 1 was a 27 year old woman who had a CD4 count of 65 at time of acute measles. MIBE onset occurred 3 months after acute measles. Measles virus PCR was positive on brain biopsy tissue. Brain virus was compared with acute virus from blood taken 3 months previously.

Patient 2 was a 14 year old boy with a CD4 count of 1 at time of neurological presentation. There was no history of a rash. This patient had received measles vaccine (Schwartz strain) during a school mass vaccination campaign some months before clinical presentation. Measles virus PCR was positive in CSF.

Patient 3 was a 34 year old woman who developed typical MIBE symptoms 3 weeks after acute measles infection. Her CD4 count at presentation was 26. Measles virus PCR was positive on post mortem brain tissue. Post mortem histology confirmed MIBE.

Patient 4 was a 24 year old woman. MIBE developed 16 weeks after acute measles when the CD4 count was 11. Measles virus PCR was positive on post-mortem brain tissue.

### Patients with acute measles

Measles virus amplified from a peripheral site such as urine or blood from 5 acutely infected patients was used to create a South African (SA) consensus sequence to which the brain virus was compared.

In addition, measles virus from blood collected at the time of acute measles was available from one patient who subsequently developed MIBE (patient 1). The brain virus of this patient was compared with her own acute virus rather than the consensus sequence.

### Nucleic acid extraction

Total nucleic acid for measles virus screening was initially extracted using the Easymag automated extractor (BioMerieux, Marcy l’Etoile, France) as per manufacturer’s instructions. Subsequently total nucleic acid was extracted from the brain or CSF using the manual Qiagen DNA mini kit with appropriate buffers for tissue and CSF extraction as per manufacturer’s instructions (Qiagen, GmbH, Germany). Nucleic acid was eluted in 50 ul elution buffer and stored at -80°C until required.

### cDNA synthesis and PCR

RNA was converted into cDNA using the RevertAid First Strand cDNA synthesis kit (Fermentas Life Sciences) and random hexamers. Briefly 11 μl RNA was incubated with 1 μl random hexamers supplied with kit at 80°C for 3 minutes and then cooled to 37°C before the addition of 4 μl 5× reaction buffer, 1 μl Ribolock RNase inhibitor (20 U/μl), 2 μl 10 mM dNTP mix and 1 μl RevertAid M-MuLV reverse transcriptase (200 U/μl) in a final 20 μl reaction volume. The mixture was incubated at 37°C for 90 min and the reaction terminated by heating to 70°C for 5 minutes. cDNA was stored at -20°C until required.

The complete nucleoprotein (N), matrix (M), fusion (F) and haemagglutinin (H) genes were amplified with primers described by Tillieux *et al*. [21]. In cases where there was no amplification after one round of PCR, nested primers designed for this study were used. (Additional file 1: Table S1).

Measles virus from a patient with acute measles was used as the positive control for all the PCR assays. PCR amplicons were generated using 6μl cDNA and Supertherm Taq DNA polymerase (JMR Holdings, UK) with the following PCR cycling conditions: an initial denaturation step of 3 min at 95°C, followed by 40 cycles of amplification (15 sec at 94°C, 30 s at 50°C and 45 s 72°C) followed by 7 min at 72°C. The nested PCR was performed using the same cycling conditions but with an increase in the annealing temperature to 55°C and 3 μl outer product.

PCR products were electrophoresed through 2% agarose gel and visualized by ethidium bromide staining and UV illumination.

### Sequencing and phylogenetic analysis

PCR products were sequenced directly in both directions with primers used for PCR amplification. The BigDye terminator cycle sequencing kit was used (Applied Biosystems, Foster City CA, USA). Sequences were assembled and aligned against the measles virus genotype B3 reference sequence (accession number HM439386) using DNA Baser Sequence Assembler v3.5.0.

The 3′ hypervariable N and Hgenes were aligned with reference sequences from GenBank using ClustalW and neighbor-joining phylogenetic trees constructed using MEGA version 5 with 1000 bootstrap re-samplings [22]. Similar neighbor-joining phylogenetic trees were also constructed for the F and M genes as well as a concatenated tree of all genes sequenced.

### Ethical approval

This study was approved by the Human Research Ethics Committee of the University of Cape Town. (HREC REF: 163/2011).

### Availability of supporting data

The data supporting the results of this article is included within the article (and its additional file(s). The local measles virus sequences were deposited in GenBank. [Gen Bank: KC305651-KC305689].

## Competing interests

None of the authors have any competing interests.

## Authors’ contributions

DH: conceived the study, analysed the results and wrote the manuscript. JH and CA: identified the cases of MIBE and helped to write the manuscript. HS: designed primers to amplify measles virus genes; assembled contigs of the genes; performed phylogenetic analysis and wrote part of the manuscript. All authors read and approved the final maunscript.

## Supplementary Material

Additional file 1: Table S1 Primer sequences used in amplification of measles virus nucleocapsid, matrix, fusion and haemaglutinin genes. Click here for file
