# Supplementary material for: Molecular characterisation of virus in the brains of patients with measles inclusion body encephalitis (MIBE)
Source: Virol J. 2013 Sep 12;10:283. doi: 10.1186/1743-422X-10-283 (PMC3847183; doi:10.1186/1743-422X-10-283)
Supplement: Additional file 1: Table S1 — Primer sequences used in amplification of measles virus nucleocapsid, matrix, fusion and haemaglutinin genes. [file 1743-422X-10-283-S1.doc]

| Name | Position | Gene | Sequence 5’-3’ |  |
| --- | --- | --- | --- | --- |
| MVNF6 | 1F | N | TCAGTACTGGACCAAACAAAGTTGGGTA | Tillieux et al.,2009 |
| MVN 970R | 970R | N | TCATGCAGTCCAAGAGCAGG | This study |
| MVN 68Fn | 68F | N | CCTATCATCAGGGACAAGAGC | This study |
| MVN 918Rn | 918R | N | GGCTAATCCTGCCTCTACGA | This study |
| MVN 840F | 839F | N | GGGAACAAACCCAGGATTGCT | This study |
| MVN 1804R | 1804R | N | GCTCCGATCGTGGGAGTGGA | This study |
| MVN 889Fn | 889F | N | TCGTAGAGGCAGGATTAGCCA | This study |
| MVN 1768Rn | 1768R | N | GGCTGTGTGGACCTGGTTCC | This study |
| MVM 3011F | 2987F | P | TGAAACCCATCATAAGTAGAGATTC | This study |
| MVM 3913R | 3941R | M | GTTATCCGAAAGACGGGTGATGCTCATAT | This study |
| MVM 3011Fn | 3007F | P | GATTCAGGCCGAGCACTGGCCG | This study |
| MVM 3913Rn | 3917R | M | CATATAAACAACACGGAACCTCTG | This study |
| MVM 3913F | 3913F | M | ATATGAGCATCACCCGTCTTTCGGATAACG | Tillieux et al., 2009 |
| MVM 4799R | 4799R | M | CTTGTGCGGTTCAGTTGTGGGGTTCTT | Tillieux et al.,2009 |
| MVM 3913Fn | 3938F | M | TAACGGGTATTACACCGTTCCTAG | This study |
| MVM 4799Rn | 4778R | M | GTTCTTGTGTTGGGGGAGG | This study |
| MVM 353F | 3737F | M | TGTTAGACGTACAGCAGGGCTC | This study |
| MVM 745R | 4150R | M | TTGCAATAATCAGCAGAGTAGAC | This study |
| MVM 385Fn | 3768F | M | CTGGTGTTCTACAACAACACTCC | This study |
| MVM 714Rn | 4098R | M | TCTTTCTCCTGAAGTTCCCGAT | This study |
| MMF4 | 4848F | M | CTCTCCCCGGCAAACTAAACAAAAC | Tillieux et al., 2009 |
| MMR6 | 5401R | M | GGAGTTGAGTGTCGTCGGGTGTGGTGGATT | Tillieux et al., 2009 |
| 2480Fn | 4861F | M | ACTAAACAAAACTTAGGGCCAAGG | This study |
| 2481Rn | 5360R | M | GTGTGGTGGATTGATCTTTTGGTC | This study |
| 2482F | 5372F | M | AATCCACCACACCCGACGACACTCAACTCC | This study |
| MFR2 | 6057R | F | GGAGTTGAGTGTCGTCGGGTGTGGTGGATT | Tillieux et al., 2009 |
| 2482Fn | 5387F | M | ACGACACTCAACTCCCCAC | This study |
| 2483Rn | 6019R | F | CAAGATAGTTGGTTCATAGACGG | This study |
| 2484F | 6028F | F | AACCAACTATCTTGTGATTTAATCGGCCAG | This study |
| MFR3 | 6579R | F | AGACCCGGATACGAGTGTACGAGCACAGGA | Tillieux et al., 2009 |
| 2484Fn | 6043F | F | GATTTAATCGGCCAGAAGCTAGG | This study |
| 2485Rn | 6542R | F | TGTACGAGCACAGGACTTGGTGG | This study |
| 2486F | 6550F | F | TCCTGTGCTCGTACACTCGTATCCGGGTCT | This study |
| MHF1 | 7000F | F | TGTTGCTGCAGGGGGCGTTGTA | Tillieux et al., 2009 |
| MHR4 | 7538R | H | CCTTGACCTGATGCTCGATTGAGTTAGTTA | Tillieux et al., 2009 |
| 2486Fn | 6568F | F | GTATCCGGGTCTTTTGGGAA | This study |
| 2487Fn | 7014F | F | GCGTTGTAACAAAAAGGGAG | This study |
| 2488Rn | 7511R | H | CGATTGAGTTAGTTACATCTAG | This study |
| MHF2 | 7471F | H | CGCAGAGATCCATAAAAGCCTCAGCACCAA | Tillieux et al., 2009 |
| MHR2 | 8150R | H | GCTCCCCCAAAGCCACCATACAGTTGC | Tillieux et al., 2009 |
| 2489Fn | 7489F | H | CCTCAGCACCAATCTAGATGTAAC | This study |
| 2490Rn | 8135R | H | CCATACAGTTGCTGAAATCATTACT | This study |
| 2491F | 8124F | H | GCAACTGTATGGTGGCTTTGGGGGAGC | This study |
| MHR3 | 8579R | H | GTGTGATCAATGGCCCGAATCC | Tillieux et al., 2009 |
| 2491Fn | 8145F | H | GGGAGCTTAAATTCGCAGCC | This study |
| 2492Rn | 8570R | H | ATGGCCCGAATCCTGAAGCAAT | This study |
| MHF5 | 8500F | H | CGGGGTCTTGTCTGTTGATCTGAGTCTGACAGTT | Tillieux et al., 2009 |
| MHR1 | 9231R | H | CGGGGAACCACTTGGACCCTACGTTTTTCT | Tillieux et al., 2009 |
| 2493Fn | 8518F | H | TCTGACTCTGACAGTTGAGCTTAAAATC | This study |
| 2494Rn | 9219R | H | TGGACCCTACGTTTTTCTTAATTCTGAT | This study |
| MLF1 | 9018F | L | GTGTGCTTGCGGACTCAGAATCTGGTGGACATA | Tillieux et al., 2009 |
| 2496R | 9583R | L | TACAGCGAATTCCCCTTTTTGAGGAGTTCACGGAT | This study |
| 2495Fn | 9033F | L | CAGAATCTGGTGGACATATCACTCACTCTGG | This study |
| 2496Rn | 9564R | L | TTGAGGAGTTCACGGATCTTCCTTGTTGAC | This study |

**Table 1S.**  Primer sequences used in amplification of measles virus nucleocapsid, matrix, fusion and haemaglutinin genes
